# Cognitive performance in hospitalized patients with severe or extreme anorexia nervosa

**DOI:** 10.1007/s40519-023-01585-w

**Published:** 2023-10-21

**Authors:** Simone Daugaard Hemmingsen, Mia Beck Lichtenstein, Magnus Sjögren, Claire Gudex, Pia Veldt Larsen, René Klinkby Støving

**Affiliations:** 1 Centre for Eating Disorders, Odense University Hospital, Odense, Denmark; 2 Research Unit for Medical Endocrinology, Odense University Hospital, Odense, Denmark; 3 Research Unit, Child and Adolescent Psychiatry, Mental Health Services in the Region of Southern Denmark, Odense, Denmark; 4 Department of Clinical Research, University of Southern Denmark, Odense, Denmark; 5 Open Patient Data Explorative Network (OPEN), Odense, Denmark; 6 Centre for Digital Psychiatry, Region of Southern Denmark, Odense, Denmark; 7 Institute for Clinical Science, Department of Psychiatry, Umeå University, Umeå, Sweden; 8 Mental Health Services in the Region of Southern Denmark, Vejle, Denmark

**Keywords:** Anorexia nervosa, Neuropsychology, Malnutrition, Eating disorder, Cognitive flexibility

## Abstract

**Purpose:**

Severe malnourishment may reduce cognitive performance in anorexia nervosa (AN). We studied cognitive functioning during intensive nutritional and medical stabilization in patients with severe or extreme AN and investigated associations between weight gain and cognitive improvement.

**Methods:**

A few days after admission to a specialized hospital unit, 33 patients with severe or extreme AN, aged 16–42 years, completed assessments of memory, cognitive flexibility, processing speed, and attention. Mean hospitalization was 6 weeks. Patients completed the same assessments at discharge (*n* = 22) following somatic stabilization and follow-up up to 6 months after discharge (*n* = 18).

**Results:**

The patients displayed normal cognitive performance at admission compared to normative data. During nutritional stabilization, body weight increased (mean: 11.3%; range 2.6–22.2%) and memory, attention, and processing speed improved (*p* values: ≤ 0.0002). No relationship between weight gain and cognitive improvement was observed at discharge or follow-up.

**Conclusions:**

Cognitive performance at hospital admission was normal in patients with severe or extreme AN and improved during treatment although without association to weight gain. Based on these results, which are in line with previous studies, patients with severe or extreme AN need not be excluded from cognitively demanding tasks, possibly including psychotherapy. As patients may have other symptoms that interfere with psychotherapy, future research could investigate cognitive functioning in everyday life in patients with severe AN.

*Trial registration number:* The study is registered at clinicaltrials.gov (NCT02502617).

**Level of evidence:**

Level III, cohort study.

**Supplementary Information:**

The online version contains supplementary material available at 10.1007/s40519-023-01585-w.

## Introduction

Anorexia nervosa (AN) is defined by a drive for thinness, restrictive eating, and low body weight. Impairment of executive functions has been suggested as a characteristic cognitive profile of patients with AN [1, 2]. Previous research has found that adults with AN have reduced cognitive flexibility (i.e., the ability to shift between different strategies) compared to healthy individuals [3–5]. However, children and adolescents with AN seem to have normal cognitive flexibility [3, 6]. A meta-analysis by Stedal et al. found reduced overall neuropsychological performance in adults with AN compared to healthy individuals [7].

Low body weight in patients with AN may affect neuropsychological performance. In line with a former meta-analysis [8], Stedal et al. (2021) found that lower BMI in adults with AN was associated with more severely impaired cognitive performance (memory, inhibition, and visuospatial abilities) [7]. Research in patients with extremely low weight and enduring AN is remarkably sparse [9], however, and it is unknown whether reduced neuropsychological performance in adults with AN is caused by undernutrition during adolescence [10], or whether the underperformance, including cognitive inflexibility, has been present before onset of the eating disorder [1].

In the absence of prospective studies with premorbid data, a number of studies have examined whether cognitive performance improves following weight gain. Some studies report improvement of at least one cognitive parameter [11–13], while others found no significant improvement [14–17]. Various neuropsychological instruments have been used to assess cognitive performance, and not all longitudinal studies accounted for practice effects [10], i.e., the improvement in scores over time due to memory for specific test items or learned strategies [18]. Measured changes in neuropsychological performance should reflect true changes in the construct being measured, so practice effects need to be taken into account when interpreting longitudinal changes [18].

Some neuropsychological tests use a parallel (different) version of the test for the second assessment, but this does not exclude the possibility of a practice effect. If the second version differs in item difficulty, it may also affect retest scores [18]. Another way of addressing a practice effect is to estimate it and adjust for it as a confounder. However, this may be difficult to do reliably in patients with severe AN. A randomized controlled trial directly investigating the effect of weight gain on cognitive performance is hardly feasible in severe AN, and these patients typically have unstable weight. During hospitalization, the weight should increase, but even in patients with unchanged weight, the hospital environment and other interventions may affect cognitive performance. A third approach has been used, where improvement in cognitive performance is examined in patients with AN compared to improvement in performance for healthy control participants [19]. Practice effects may differ in populations of patients with AN and healthy participants, however.

A fourth approach to dealing with practice effects is to focus on the association between change in cognitive performance and proportional change in weight during hospitalization while controlling for baseline cognitive performance. The possibility of a practice effect on cognitive improvement would be present for all participants, but it would have lower impact on the association with weight gain. As no previous study has taken this approach [10], it remains unknown whether cognitive functions improve with weight restoration treatment in adults with severe AN, and it could be discussed whether weight gain should be the priority during hospitalization over other measures of outcome.

NICE guidelines on treating AN (www.nice.org.uk/guidance, Sections 1.3, last updated 16 December, 2020), state that “weight gain is key in supporting other psychological, physical and quality of life changes that are needed for improvement or recovery.” (1.3.2). However, whether weight gain is needed to obtain normal cognitive functioning and partake in cognitively demanding tasks is unknown.

The aim of the current prospective study was to investigate whether adult patients with severe or extreme AN had cognitive difficulties compared to the general population and whether any changes in cognitive performance during treatment were associated with weight gain.

The specific objectives were thus (i) to investigate whether patients with severe or extreme AN on admission to a somatic stabilization unit had specific cognitive difficulties identified through a broad test battery (including tests of memory, processing speed, attention, and cognitive flexibility) compared to the normative population for age groups, (ii) to investigate whether their cognitive performance improved during hospitalization, (iii) to investigate association between proportional weight change and absolute change in Trail Making Test switching performance during hospitalization, (iv) to investigate any associations between proportional weight change and absolute change in other cognitive performance measurements during hospitalization, and (v) to explore any associations between proportional weight change and absolute change in cognitive performance measurements from discharge to follow up 2–6 months after discharge.

## Methods

### Study description

The cohort study was conducted at the Center for Eating Disorders, Odense University Hospital, and the Mental Health Services in the Region of Southern Denmark. In addition to the investigation of cognitive performance, the cohort study investigated cortisol levels, depression, and anxiety in patients with severe AN [20]. Patients (≥ 16 years) admitted to the specialized nutrition unit for eating disorders at Odense University Hospital between March 2016 and July 2021 were invited to participate. Participants were excluded if they had schizophrenia, other psychotic disorders, or active substance abuse; if their pharmacological treatment changed during re-nutrition; if they could not read Danish; or if they were admitted primarily for short-term (1–3 days) fluid and electrolyte correction (as assessed by the chief physician).

### Recruitment and participants

**Fig. 1:**
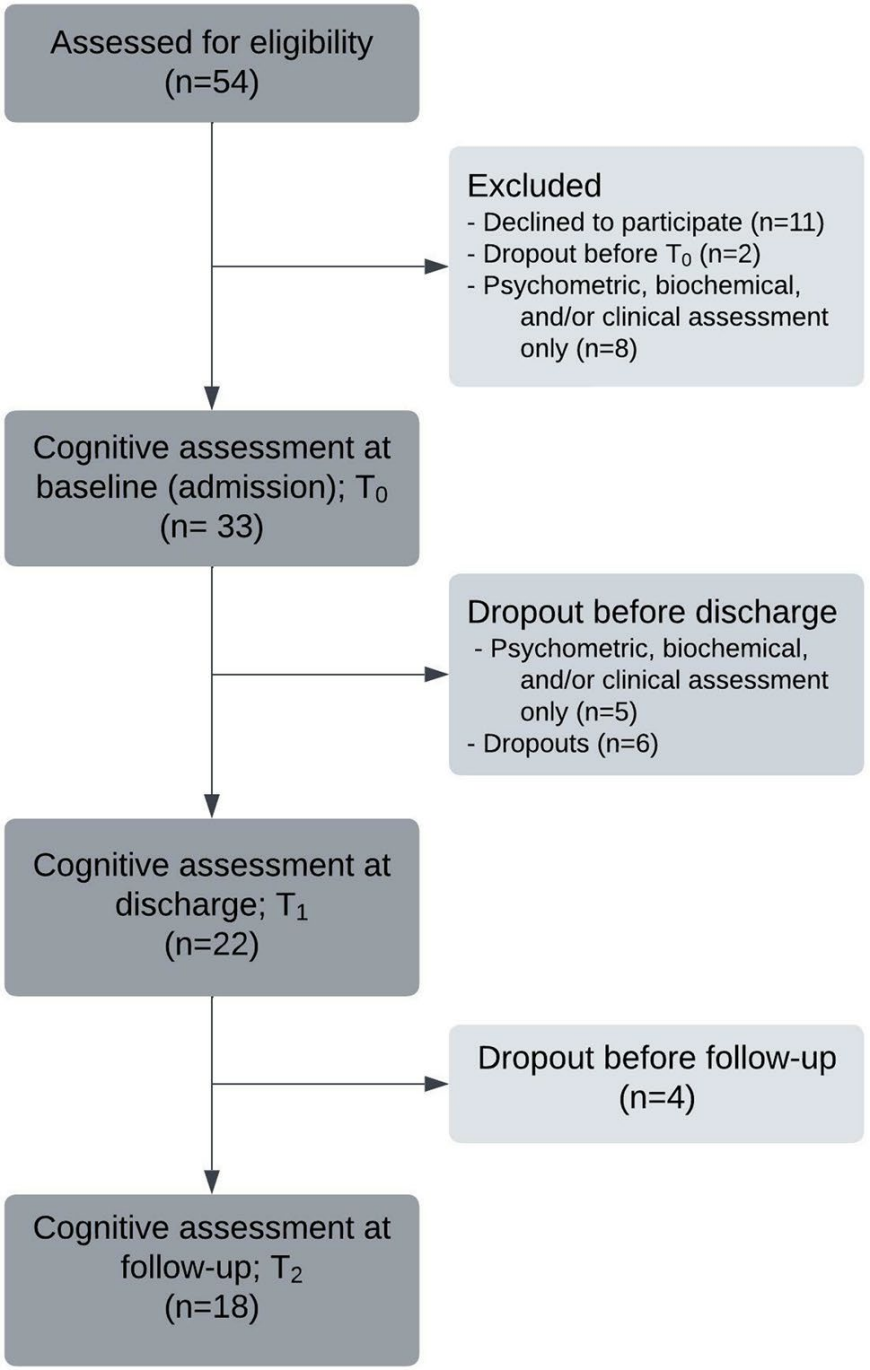
Flowchart of the study

The study comprised three assessments (1) at admission (*T*_0_), (2) at discharge (*T*_1_), and (3) at 2–6 month follow-up (*T*_2_). A flowchart of the study is presented in Fig. 1. We invited 54 patients to participate. Of these, 41 agreed to participate and 33 patients completed the cognitive assessment (full test battery) at admission. We included patients from across the country, but most lived in the Region of Southern Denmark. We included patients regardless of their race, ethnic origin, gender, sex, sexual orientation, and socioeconomic status.

The sample consisted of 32 females and one male with AN, aged 16–42 years. Patients were diagnosed according to the Diagnostic and Statistical Manual of Mental Disorders fifth edition (DSM-5) [21]. The included patients were categorized as suffering from severe or extreme AN according to the DSM-5. A total of 27 patients (82%) had extreme AN (BMI below 15), and six patients had severe AN (BMI 15 or above). All included patients were assessed by the chief physician as having a need for specialized nutritional treatment and were admitted due to functional disability, dizziness, and bradycardia following rapid weight loss and/or purging behavior.

### Re-nutrition intervention

The intervention for patients with severe or extreme AN at the specialized nutrition unit is short-term intensive nutritional, somatic, and medical stabilization. The cognitive assessment was conducted during treatment as usual in the unit. Following re-hydration and individualized up-titration in energy intake according to guidelines [22], the treatment goal is safe and effective weight gain of 2–3% per week. The daily energy intake is, therefore, highly individualized according to the weight course. If a patient failed to reach 2% weekly weight gain, the energy level of the menu was increased. Meals were supervised by trained nurses at scheduled times and were followed by a 1-h supervised rest in a seated position. The macronutrient proportions for meals were 40–50% carbohydrate with maximum 10% sugar; 30–40% fat; and 20–25% protein. The re-nutrition program during hospitalization is described in detail in [20].

### Data collection

We used Research Electronic Data Capture (REDCap) hosted at Odense Patient data Explorative Network (OPEN) for the data collection. REDCap is a secure web platform for surveys and databases [23, 24]. Data were collected from clinical assessments, psychometric self-report questionnaires, and cognitive assessments undertaken during treatment as usual in the nutrition unit. Assessments at admission (*T*_0_) were conducted after approximately 1 week—and no earlier than 3 days—after hospitalization to ensure acclimatization and stabilization of liquid–electrolyte balance, vitamins and minerals, hypoglycemia, infections, and other intercurrent somatic diseases, all of which could potentially influence cognitive performance. Discharge measurements (*T*_1_) were conducted a few days before discharge. However, three participants were assessed with the cognitive tests a few days after discharge, because they dropped out of treatment. Follow-up measurements (*T*_2_) were conducted a couple of months after discharge (*T*_1_), but in some cases up to 6 months after discharge. Six patients were re-hospitalized at follow-up (*T*_2_).

### Reducing risk of bias

It was not possible to use blind assessment. However, the clinical measurements and collection of clinical data from the patient records were collected by a medical doctor (RKS), while two psychologists (SDH and FP) administered the neuropsychological test battery. The same psychologist (SDH) scored all tests to ensure homogeneity in scoring. Scoring took place after collection of data for all three timepoints, or after the patient had dropped out. Assessments during hospitalization were conducted in the patient’s room after either breakfast (assessment at 8.30–10.30) or lunch (assessment at 12.30–14.30).

### Measurements

#### Clinical measurements and medical records

Anthropomorphic measurements were conducted by specialized nurses in the nutrition unit on the day of cognitive assessments. Weight was measured on a calibrated platform scale, and height was measured on a wall-mounted stadiometer. BMI was calculated as weight divided by height (kg/m^2^). Age, illness duration, nadir BMI (lowest BMI registered), and co-morbidity were collected from the patient’s hospital medical records. Educational level was collected via interview.

#### Psychometric measurements

The following psychometric questionnaires were distributed via e-mail at all three timepoints for the patients to complete in privacy:

Eating Disorder Inventory 3 (EDI-3) [25] in Danish [26], Toronto Alexithymia Scale TAS-20 [27] in Danish [28], Beck Depression Inventory II (BDI-II) [29] in Danish (Pearson Assessment, Danish version from 2005), Hospital Anxiety and Depression Scale (HADS) [30] in Danish [31], and Perceived Stress Scale 10 (PSS-10) [32] in Danish [33]. If patients did not complete the questionnaires within a few days, e-mail reminders were sent via REDCap.

#### Cognitive performance

We selected a test battery of validated neuropsychological tests in collaboration with an experienced neuropsychologist. The tests were chosen to investigate cognitive functions that were experienced to be affected in patients with severe or extreme AN—either by health care professionals working in the specialized nutrition unit, by the patients in the unit (memory functions, processing speed, and attention), or by previous studies in patients with AN (cognitive flexibility [3] and memory [34]). We used the Danish versions of the tests, except for the Wechsler Memory Scale III for which we used the Swedish/Norwegian version. The test battery was administered in a fixed order and took approximately 2 h. The following tests were administered in the patient’s room or a consultation room by a trained psychologist or trained psychology master student at admission (*T*_0_), discharge (*T*_1_), and follow-up (*T*_2_):

- ***The Wechsler memory scale III* (WMS-III)** [35] We administered the following subtests: Logical memory I and II, Verbal paired associates I and II, Faces I and II, Family pictures I and II, Letter-number sequencing, and Spatial span. The subtests were converted into the following primary indexes: (1) Auditory Immediate Index and Auditory Delayed Index (measurements of auditory memory), (2) Visual Immediate Index and Visual Delayed Index (measurements of visual memory, (3) Immediate Memory Index (global measurement of immediate memory, including both auditory and visual memory), (4) Auditory Recognition Delayed Index (the recognition counterpart to the Auditory Delayed Index, which incorporated a recall procedure), (5) General Memory Index (global measurement of delayed memory, including both auditory and visual memory), and (6) Working Memory Index (including both auditory and visual memory).
- ***The d2 test of attention—revised*** [36, 37] measuring attention and concentration performance.
- ***The processing speed index (PSI)*** of the Wechsler Adult Intelligence Scale IV (WAIS-IV)[38]. The subtests Symbol Search and Coding were administered.
- ***Verbal fluency test, design fluency test, and trail making test*** of the Delis–Kaplan Executive Function System (D-KEFS)[39] measuring cognitive flexibility. The Trail Making Test switching condition is of primary interest considering previous research and is, therefore, the primary outcome. More detailed descriptions of the neuropsychological test battery, i.e., test items, internal consistency, and test–retest reliability, are provided in Table 2 in [40].

### Pilot study and sample size considerations

A pilot study was conducted with five patients prior to data collection to ensure study feasibility and relevance.

Only one previous study has investigated the association between change in cognitive performance and weight change in the same age group as in the current study [41], and they used a different set of cognitive tests than in the current study. In the absence of previous results to inform a power calculation, sample size was based on the recommendation by Jenkins and Quintana-Ascencio that ≥ 25 participants should be included in research based on regression models [42]. They investigated the minimum *N* to identify plausible data patterns due to concerns about the reproducibility of research with small sample sizes, which is common in some clinical areas. Jenkins and Quintana-Ascencio found that a minimum *N* of 8 was required for small variance, and a minimum of 25 was required for larger variance. Therefore, we aimed for a sample size of 25 participants. Expecting a dropout rate of 20%, we included 33 patients at baseline.

### Statistics

There were no missing data. STATA 17 was used for all statistical analyses. Number, mean, standard deviation, and range are presented for continuous variables and number of participants and percentage for categorical variables.

### Primary analysis

Relationship between proportional weight change and absolute change in Trail Making Test switching condition scores from admission (*T*_0_) to discharge (*T*_1_) was calculated with a linear regression analysis, adjusting for baseline values of cognitive performance.

### Secondary analyses

To account for multiple testing, we used Šidák corrected significance levels for each table.

- Paired *t* tests were used to examine differences between admission (*T*_0_) and discharge (*T*_1_) cognitive performance raw scores and indexes. Cohen’s d effect sizes were calculated.
- Comparisons between cognitive performance at admission and normative data were calculated with one sample *t* tests between sample means and normative data for the general population from test manuals. Cohen’s d effect sizes were calculated.
- Relationships between proportional weight change and absolute change in cognitive performance for the WMS-III indexes and subtasks, the WAIS-IV processing speed, the D-KEFS tests, and the d2 test of attention from admission (*T*_0_) to discharge (*T*_1_) and from discharge (*T*_1_) to follow up (*T*_2_) were calculated with linear regression analyses, adjusting for baseline values of cognitive performance.
- Linear regression analyses as described in c) were performed, adjusting further for time interval between *T*_0_–*T*_1_ and *T*_1_–*T*_2_, respectively.
- Linear regression analyses as described in c) were performed, adjusting further for depression scores.
- Linear regression analyses as described in c) were performed, adjusting further for illness duration.

Histograms of the empirical distributions of the WMS-III and the WAIS-IV indexes and the D-KEFS switching tasks were produced overlaid with the normative distributions. The WMS-III and the WAIS-IV indexes are normally distributed with a mean of 100 and a standard deviation of 15. The D-KEFS scaled scores have a mean of 10 and a standard deviation of 3.

### Sensitivity analyses

Sensitivity analyses of all above analyses were performed, excluding one patient who was 16 years (and leaving patients $\ge$ 18 years), because age might play a role in the cognitive profile of patients with AN. Furthermore, linear regression analyses were used to examine cognitive differences at baseline between patients with the restrictive subtype of AN and patients with the binge/purge subtype of AN.

### Dropout analysis

A dropout analysis comparing patients who completed the discharge assessment (*T*_1_) and patients who dropped out before the discharge were conducted using c^2^-tests (categorical covariates), Wilcoxon ranksum tests (non-normally distributed continuous covariates), and *t* tests (normally distributed covariates).

### Ethics

The study was carried out in accordance with the latest version of the Declaration of Helsinki. The study was approved by the Regional Research Ethics Committees of Southern Denmark (project ID S-20150042) and the Danish Data Protection Agency. We registered the study at clinicaltrials.gov (NCT02502617) prior to data collection. Participants (and their parents when applicable) provided written informed consent. At an informational meeting, patients received study information and the purpose of the research project was described.

### Data availability

The data that support the findings of this study are available in an anonymized version from the corresponding author upon reasonable request. The materials used are licensed (references in text).

## Results

### Sample characteristics

During the pilot study and main data collection, we found that the patients were able to hold their concentration during assessments, but they expressed that they were tired afterwards, and some were too physically weak to sit up during assessments. There were no significant baseline differences on cognitive or demographic variables between patients who dropped out after the admission assessment (*T*_0_) and patients who completed the discharge assessment (*T*_1_). Overall, the patients gained 11.3% body weight during hospitalization. Between discharge (*T*_1_) and follow-up (*T*_2_), 50% of the patients lost weight (up to 17% of body weight) and 50% gained weight (up to 27.6% of body weight), with an average gain of 2% body weight. At discharge, none of the patients had BMI > 18.5, and at follow-up, three patients had BMI > 18.5. At admission, 82% (*n* = 27) of the patients were in the category of extreme AN (BMI < 15). A total of 16 patients had an admission BMI > 13.

Demographic and admission characteristics are presented in Table 1.

**Table 1:** Demographic and clinical characteristics of the study sample of patients with severe anorexia nervosa

	*n* (%)	Mean (SD)	Range
**Total**	33 (100)	–	
**Sex**			
Female	32 (97)	–	
**Severity of AN according to DSM-5**			
Severe (BMI ≥ 15)	6 (18)	–	
Extreme (BMI < 15)	27 (82)	–	
**Diagnostic subtype**			
Restrictive	19 (58)	–	
Binge/Purging	14 (42)	–	
**Co-morbidity**			
None	26 (79)	–	
Personality disorder, OCD, Autism spectrum disorder	7 (21)	–	
**Age, years**	33 (100)	26.1 (7.5)	16–42
**Illness duration, years**	33 (100)	8.0 (6.3)	1–21
**Education, years**	33 (100)	12.0 (2.2)	8–18
**Nadir weight, kg**	33 (100)	32.8 (7.0)	18–46
**Nadir BMI**, **kg/m**^**2**^	33 (100)	12.1 (2.2)	7.5–17.0
**Weight change 4 weeks prior to admission **(*T*_0_), %	33 (100)	–	− 32–15
**Weight loss (≥ 5%)**	13 (39)	–	− 32–(− 5.8)
**Weight stable (< 5% weight change)**	14 (43)	–	− 3.9–3
**Weight gain (≥ 5%)**	6 (18)	–	5–15
**Admission** (*T*_0_) **BMI,** **kg/m**^**2**^	33 (100)	13.2 (2.2)	8.4–18.1
**Discharge **(***T***_**1**_) **BMI**, **kg/m**^**2**^	22 (67)	14.3 (1.8)	9.3–17.9
**Follow-up** (***T***_**2**_) **BMI**, **kg/m**^**2**^	18 (55)	14.8 (3.1)	7.7–19.5
**Weight gain during hospitalization, kg**	22 (67)	3.7 (1.5)	0.9–6.5
**Weight gain during hospitalization, %**	22 (67)	11.3 (5.4)	2.6–22.2
**Weight change during follow-up period, kg**	18 (55)	1.1 (5.1)	− 6.8–12.3
**Weight change during follow-up period, **%	18 (55)	2.0 (12.7)	− 17.0–27.6
**Weeks between admission **(***T***_**0**_) **and discharge** (*T*_1_)	22 (67)	6.3 (3.0)	1–13
**Weeks between discharge** (***T***_**1**_) **and follow-up** (*T*_2_)	18 (55)	23.8 (13.0)	2–49
**Psychometrics at admission** (***T***_**0**_)			
PSS-10	33 (100)	27.6 (5.7)	16–37
BDI-II	33 (100)	34.1 (11.0)	10–52
TAS-20	33 (100)	57.1 (12.0)	29–76
HADS anxiety	33 (100)	13.7 (4.0)	2–19
HADS depression	33 (100)	10.3 (4.4)	3–19
EDI-3			
*Drive for thinness*	33 (100)	19.0 (8.1)	0–28
* Bulimia*	33 (100)	7.3 (8.1)	0–27
*Body dissatisfaction*	33 (100)	27.5 (11.2)	0–40
**Psychometrics at discharge **(***T***_**1**_)*			
PSS-10	21 (64)	26.5 (6.2)	16–36
BDI-II	21 (64)	30.3 (10.5)	9–48
TAS-20	21 (64)	54.8 (11.0)	34–71
HADS anxiety	21 (64)	13.3 (3.5)	5–20
HADS depression	21 (64)	10.1 (4.3)	3–21
EDI-3			
*Drive for thinness*	21 (64)	19.0 (8.2)	0–28
* Bulimia*	21 (64)	6.3 (7.0)	0–21
* Body dissatisfaction*	21 (64)	27.6 (10.2)	5–40
**Psychometrics at follow-up** (***T***_**2**_)*			
PSS-10	17 (55)	27.4 (5.7)	18–39
BDI-II	17 (55)	29.8 (12.0)	5–50
TAS-20	17 (55)	54.3 (13.1)	29–71
HADS anxiety	17 (55)	12.9 (4.2)	2–18
HADS depression	17 (55)	9.9 (4.4)	2–19
EDI-3			
*Drive for thinness*	17 (55)	19.1 (8.0)	0–28
* Bulimia*	17 (55)	4.2 (5.5)	0–21
*Body dissatisfaction*	17 (55)	27.5 (10.8)	5–40

Admission (*T*_0_) measurements were conducted on the day of cognitive assessments (no earlier than 3 days after hospital admission)

*SD* standard deviation, *DSM-5* Diagnostic and Statistical Manual of Mental Disorders fifth edition, *BMI* Body Mass Index, *OCD* obsessive–compulsive disorder; *nadir* lowest lifetime weight and/or BMI, *PSS-10* Perceived Stress Scale 10, *BDI-II* Beck Depression Inventory II, *TAS-20* Toronto Alexithymia Scale-20, *HADS* Hospital Anxiety and Depression Scale, *EDI-3* Eating Disorder Inventory 3

*One of the patients did not complete the questionnaires at discharge nor at follow-up

### Cognitive improvement

Table 2 shows cognitive raw scores and indexes at admission and discharge. All memory indexes of the WMS-III, except for working memory, improved during hospitalization. Processing speed of the WAIS-IV improved. Attention of the d2 test and of two out of three conditions of the Trail Making Test improved. None of the effect sizes exceeded two standard deviations. Improvement on the cognitive flexibility measurements of the Verbal Fluency Test, the Design Fluency Test, and the Trail Making Test during hospitalization was not significant.

**Table 2:** Cognitive improvement from admission (*T*_0_) to discharge (*T*_1_) for the 22 patients with anorexia nervosa completing the discharge assessment

	Admission		Discharge		*p* values	Cohen’s *d*
	*M*	SD	*M*	SD		
**D-KEFS**						
**Trail making test**						
Condition 1: Visual search, raw score	20.57	6.27	18.32	5.80	0.0218	− 0.53
Condition 2: Numbers, raw score	31.73	12.11	26.36	11.85	< 0.0001*	− 1.09
Condition 3: Letters, raw score	30.97	10.14	24.77	8.76	0.0003*	− 0.92
Condition 4: Number− letter, raw score	75.18	22.04	62.55	22.21	0.0139	− 0.57
Condition 4: Errors	0.68	0.83	0.55	0.86	0.5441	− 0.13
Condition 5: Motor speed, raw score	25.66	9.43	23.27	7.56	0.0284	− 0.50
**Verbal fluency test**						
Condition 1: Phonemic fluency, raw score	35.86	16.24	41.05	14.93	0.0036	0.70
Condition 2: Semantic fluency, raw score	44.23	9.58	46.41	10.28	0.1022	0.36
Condition 3: Category switching, raw score	15.77	2.65	16.09	3.50	0.6797	0.09
Category errors, raw score	1.05	1.65	0.82	1.01	0.5651	− 0.12
Repetition errors, raw score	1.55	3.56	1.50	2.22	0.9356	− 0.02
**Design fluency test**						
Condition 1: Filled dots, raw score	10.18	3.51	13.41	3.47	< 0.0001*	1.63
Condition 2: Empty dots, raw score	10.73	3.21	13.73	3.78	0.0002*	0.94
Condition 3: Switching, raw score	9.77	2.78	10.09	2.69	0.3903	0.19
Category errors, raw score	1.09	1.06	1.27	1.16	0.6110	0.11
Repetition errors, raw score	4.00	4.81	4.23	3.66	0.7906	0.06
**WMS-III**						
Auditory immediate index	99.91	19.96	113.95	16.73	< 0.0001*	1.61
Visual immediate index	93.50	16.48	109.95	15.90	< 0.0001*	1.43
Immediate memory index	96.18	20.93	114.64	17.78	< 0.0001*	1.60
Auditory delayed index	99.00	16.79	110.91	12.13	< 0.0001*	1.11
Visual delayed index	96.73	14.19	111.68	15.04	< 0.0001*	1.25
Auditory recognition delayed index	92.50	10.66	108.41	16.36	< 0.0001*	1.29
General memory index	95.82	15.46	113.18	14.26	< 0.0001*	1.79
Working memory index	100.09	11.99	102.68	11.66	0.1138	0.35
**WAIS-IV**						
Processing speed index	98.59	9.07	109.32	11.89	< 0.0001*	1.36
**d2-R**						
Processed targets, raw score	445.27	82.05	502.68	72.92	< 0.0001*	1.37
Errors, raw score	13.23	9.11	14.64	13.55	0.5256	0.14
Accuracy (% errors), raw score	2.89	2.01	2.85	2.48	0.9301	− 0.02
Corrected total score, raw score	432.05	81.73	488.05	69.68	< 0.0001*	1.54
Concentration performance, raw score	175.50	38.71	200.00	35.03	< 0.0001*	1.63

*SD* Standard deviation

Šidák corrected significance level: *a* = 0.00117

*Statistical significance at the corrected significance level

The 18 patients who completed the follow-up assessment showed no significant changes in cognitive performance from discharge to follow-up.

### Comparison between admission scores and normative data

Supplementary Figures S1 and S2 illustrate the sample distributions of the WMS-III and the WAIS-IV indexes and the scaled switching scores of the Trail Making Test, Design Fluency Test, and the Verbal Fluency Test of the study sample compared to the normal distribution curves of the normative data.

For the few significant differences in cognitive performance between the study data and the normative data, the patients with AN performed better than the general population (Table 3). None of the effect sizes exceeded 1 standard deviation.

**Table 3:** Comparison of cognitive performance between the 33 patients with anorexia nervosa at admission (*T*_0_) and normative data from the general population

Instrument	Study sample		*p* values	Cohen’s *d*
	Mean (SD)	95% CI		
**Trail making, scaled scores**				
Condition 1: Visual search	10.64 (2.52)	9.74–11.53	0.1570	− 0.25
Condition 2: Numbers	10.03 (3.12)	8.92–11.14	0.9558	− 0.01
Condition 3: Letters	10.12 (2.91)	9.09–11.15	0.8126	− 0.04
Condition 4: Number-letter	9.42 (2.76)	8.44–10.40	0.2399	0.21
Condition 5: Motor speed	11.09 (1.62)	10.51–11.67	0.0005 *	− 0.67
**Verbal fluency, scaled scores**				
Condition 1: Phonemic fluency	10.82 (4.86)	9.09–12.54	0.3411	− 0.17
Condition 2: Semantic fluency	12.15 (4.01)	10.73–13.57	0.0042	− 0.54
Condition 3: Category switching, responses	12.67 (2.90)	11.64–13.70	< 0.0001*	− 0.92
Condition 3: Category switching, switching	12.06 (2.97)	11.01–13.11	0.0004 *	− 0.69
Category errors	10.94 (2.41)	10.08–11.79	0.0322	− 0.39
Repetition errors	9.82 (3.37)	8.62–11.01	0.7585	0.05
**Design fluency, scaled score**s				
Condition 1: Filled dots	11.03 (3.30)	9.86–12.20	0.0825	− 0.31
Condition 2: Empty dots	10.33 (3.04)	9.26–11.41	0.5330	− 0.11
Condition 3: Switching	11.18 (3.08)	10.09–12.27	0.0346	− 0.38
Combined score	12.03 (4.86)	10.31–13.75	0.0223	− 0.42
Category errors	12.09 (2.66)	11.15–13.03	0.0001*	− 0.79
Repetition errors	10.94 (2.41)	10.08–11.79	0.0322	− 0.39
Processed targets	11.94 (3.98)	10.53–13.35	0.0085	− 0.49
Percent accuracy	9.12 (2.89)	8.10–10.15	0.0904	0.30
**WAIS-IV index score**				
Processing speed index	101.48 (12.87)	96.92–106.05	0.5124	− 0.12
**WMS-III index scores**				
Auditory immediate index	99.82 (18.59)	93.23–106.41	0.9556	0.01
Visual immediate index	93.76 (16.66)	87.85–99.66	0.0390	0.37
Immediate memory index	96.30 (20.09)	89.18–103.43	0.2985	0.18
Auditory delayed index	99.97 (15.87)	94.34–105.60	0.9913	< 0.01
Visual delayed index	96.70 (13.75)	91.82–101.57	0.1771	0.24
Auditory recognition delayed index	95.15 (11.62)	91.03–99.27	0.0226	0.42
General memory index	96.97 (15.10)	91.61–102.33	0.2577	0.20
Working memory index	99.42 (11.89)	95.21–103.64	0.7826	0.05
**d2 test of attention, percentiles**				
Processed targets	43.91 (29.47)	33.46–54.36	0.2439	0.21
Corrected total score	44.85 (29.95)	34.23–55.47	0.3305	0.17
Concentration performance	48.64 (27.67)	38.82–58.45	0.7789	0.05

*SD* standard deviation, *CI* confidence interval

Šidák corrected significance level: *a* = 0.0017

*Indicates significance

One-sample *t* tests were used to investigate differences between the study sample data and normative data. Mean normative score for WMS-III and WAIS-IV indexes is 100 (SD = 15) and for Trail Making Test, Verbal Fluency Test, and Design Fluency Test scaled scores is 10 (SD = 3). Mean normative percentile for d2 Test of Attention is 50

Study sample scores are scored in relation to age (age groups differ for each test). Information on age groups and means (SDs) are provided in the test manuals: WAIS-III–WMS-III Technical Manual [43], WAIS-IV Technical and Interpretive Manual [38], d2 Test of Attention in Danish d2 testen—en vurdering af opmærksomhed og concentration [37], D-KEFS Technical Manual [39]

### Relationship between proportional weight gain and cognitive improvement

No statistically significant relationships between proportional weight gain and cognitive improvement from admission to discharge or from discharge to follow up were found on any cognitive measurements.

Table 4 presents results of the relationship between proportional weight change and absolute change in Trail Making Test switching scores.

**Table 4:** Relationship between proportional weight change and change in cognitive performance on the Trail Making Test switching condition raw scores from admission (*T*_0_) to discharge (*T*_1_) and from discharge (*T*_1_) to follow up (*T*_2_)

Condition 4: Number-letter			
	*β* (95% CI)	*p* values	*n*
*T*_0_—> *T*_1_	− 0.032 (− 1.831–1.766)	0.970	22
*T*_1_—> *T*_2_	− 0.361 (− 1.111–0.388)	0.317	18

*CI* Confidence interval

Associations between absolute change in cognitive performance for all test scores and proportional weight change from admission (*T*_0_) to discharge (*T*_1_), and from discharge (*T*_1_) to follow up (*T*_2_) are presented in Supplementary Tables S1–S3. There were no statistically significant relationships.

Additional analyses included adjusting for illness duration, depression scores at admission, or time interval between assessments. None of these analyses yielded significant results on the relationship between proportional weight gain and cognitive improvement.

Sensitivity analyses including only patients who were ≥ 18 years did not change the results reported in Supplementary Tables S2–S4. Furthermore, there were no significant differences in cognitive performance at baseline between patients with the binge/purge subtype of AN, and the patients with the restrictive subtype.

## Discussion

Patients with severe or extreme AN demonstrated normal cognitive performance a few days after admission to a specialized nutrition unit. The patients showed improved cognitive performance during hospitalization while gaining body weight (mean 11.3%). There were no statistically significant associations between weight gain and cognitive improvement in terms of cognitive flexibility, memory, processing speed, or attention during hospitalization with partial weight restoration treatment. Nor were there any statistically significant associations between weight change and change in cognitive performance from discharge to follow up several months after discharge.

### Normal cognitive performance in severe AN

The patients with severe or extreme AN did not display cognitive impairment on the test battery used in our study. Some memory index scores were a few points lower than the normative data, but the differences were not statistically significant. Patients performed better than the normative data on one task of cognitive flexibility (verbal fluency switching), but not on other cognitive flexibility tasks. The result of good verbal cognitive flexibility in patients with AN is in line with a previous study by Stedal et al. [44]. The patients also performed better than the normative data on motor speed on the Trail Making Test and made fewer category errors on the Design Fluency Test. Overall, the patients with AN demonstrated normal cognitive performance on all measurements.

We had expected the patients with severe AN to display some cognitive difficulties upon admission. A recent systematic review found that patients with severe and enduring AN performed lower on decision-making compared to healthy controls, but results regarding intelligence and cognitive flexibility were conflicting, and the patients performed similar to healthy controls on memory, attention, central coherence, and processing speed [9]. In line with our results, Seidel et al. found no differences between patients with enduring AN and healthy controls in relation to most cognitive functions, except for faster reaction times and increased accuracy in patients with enduring AN [17]. Few studies have included patients with AN with extreme and persisting low weight, however. The study by Rylander et al. investigated severely malnourished patients with AN and found no cognitive impairment in line with the results from the current study [45]. The current sample consisted mostly of patients with extreme AN with long illness duration and frequent hospitalizations for life-saving fluid electrolyte correction and re-nutrition. However, there was large variation in illness duration (from 1 to 21 years). Adjusting for illness duration did not alter our finding of no association between weight gain and cognitive improvement.

A severe fast weight loss might affect cognitive performance differently than a severe persisting low weight over time. Cognitive adaptation to severe malnutrition may occur, where cognitive functions remain unchanged or regain normality with persisting low weight. This is supported by a case report of a patient with an illness duration of 25 years and a BMI of 7.7 who completed the test battery used in the current study [40]. In our study, 82% of patients had extreme AN according to the DSM-5 [21] severity rating based on BMI levels. Despite this, the patients demonstrated normal cognitive performance.

A previous study found that cognitive inflexibility could persist even after recovery from AN [46], but our results showed no evidence of cognitive inflexibility at hospital admission compared with normative data. It is possible that our cognitive flexibility tasks were too simple, but an earlier study in patients with AN found less cognitive flexibility than control participants on the Trail Making Test also used in the current study [47]. A recent systematic review of cognitive flexibility in AN found mixed results, but many studies found no difference in Trail Making Test switching scores between adults with AN and healthy participants [4].

The variability in study results may be due to varying study designs and small sample sizes, and possibly there is a difference between neuropsychological performance on the cognitive flexibility tests and cognitive–behavioral flexibility in everyday life [48].

Cognitive inflexibility seems to be present in patients with other eating disorders, such as binge eating disorder [49] and bulimia nervosa [50]. In our study, there were no cognitive differences at admission between patients with the binge/purge and the restrictive subtype of AN.

### Cognitive improvement and practice effects

Although scores were within the normal range, patients in our study significantly improved cognitive performance on most memory indexes, processing speed, concentration performance, and attention. We had expected this as patients and health care professionals in the nutrition unit generally observe that memory and concentration improve during hospitalization. However, we found no associations between proportional weight gain and improvement of any on the cognitive functions. Therefore, the cognitive improvement measured during treatment was possibly not clinically relevant and may have been caused by practice effects.

A systematic review suggested that children and adolescents might improve processing speed more than could be accounted for practice effects (investigated in healthy participants) following weight gain [10]. We focused on the relationship between cognitive improvement and proportional weight gain, because practice effect might have confounded the improvement in memory, attention, and processing speed in our sample. We recommend that future research on larger samples could adjust for confounders such as practice effects (measured on a sample of healthy control participants) when investigating associations between cognitive improvement and weight gain in patients hospitalized for AN.

Few studies have examined the relationship between weight gain and improvement in a range of cognitive functions in AN [12, 41, 51]. Only the study by Moser et al. was conducted among adults (participants aged 16–42 years), and none of the studies found significant associations in line with the results from our study. Sensitivity analyses to include only patients aged over 18 years or adjusting for depression, illness duration, or time interval between assessments did not change the main findings in our study.

NICE guidelines state that weight gain is important for psychological and physical improvements in AN. Patients with severe and enduring AN cannot be expected to reach a normal weight, however. For patients who have been severely ill for 8–12 year full recovery is unlikely [52]. A previous study expressed concern for these patients, because they are often excluded from psychotherapeutic treatment in the UK [53]. It has gained media attention that first priority in treatment is weight gain rather than psychotherapy. An argument against inviting severely malnourished patients to engage in psychotherapy has been that underweight impairs cognitive ability, so weight gain could reverse the effect of malnutrition. In the case of extreme and enduring AN, however, it is unlikely that the patient will reach a BMI level above 15. The current study did not find that weight gain was related to cognitive improvement during hospitalization for severe or extreme AN. Therefore, it is possible that (often chronic) patients with extreme AN should be offered psychotherapy with focus on improving quality of life instead of aiming for recovery.

## Conclusions and clinical implications

A few days after admission to a specialized nutrition unit for severe AN, following fluid and electrolyte correction, the patients in this study did not display cognitive impairment when compared to the normative means. This indicates that it is possible to use neuropsychological tests to assess hospitalized patients with severe or extreme AN even while they are extremely malnourished. There was no association between weight gain and cognitive improvement during hospitalization or follow-up. The study results are in line with those from previous studies and suggest that patients with severe AN do not need to be excluded from cognitively demanding tasks, possibly including psychotherapy, while being severely malnourished. It is possible that other symptoms, such as obsessiveness, may interfere with psychotherapy, however, and future research could investigate (ecologically valid) cognitive functioning in everyday life in patients with severe AN.

## Strengths and limitations

A strength of the study is that the cognitive assessment at admission was performed at least 3 days after admission to ensure fluid–electrolyte correction before the assessment (but before weight gain). This decision was mainly based on two factors. First, addressing the immediate physical health concerns such as dehydration, hyponatremia, and hypoglycemia helped to ensure that the patients were in a relatively stable physiological state for a more accurate cognitive assessment. Second, giving patients time to mentally acclimatize to the hospital environment before conducting cognitive tests may reduce anxiety and enhance their overall performance. This is an issue that has not received much attention in previous studies of cognitive functions in patients with severe AN.

In addition to assessing patients at admission and discharge, we assessed the patients at a follow-up several months following discharge. During this time interval, we expected many patients to lose weight (50% of the patients did lose weight), and therefore, the patients could act as their own controls. We addressed the absence of blind assessment by having the clinical data and cognitive data collected by two different researchers, and the same psychologist scored the tests after collection of data for all timepoints.

A limitation of the study was that we compared cognitive performance in patients with AN to normative data instead of to a control group. The patients with AN performed well compared to normative data, but they might have performed lower on the cognitive tests than a matched control group. The normative data sets were derived from German or American populations that might differ from the Danish population. In addition, we did not include a measure of general cognitive ability. Since there are high correlations between general cognitive ability and specific cognitive functions, this might have been a confounder, e.g., if low general cognitive ability led to poor memory. In the current sample, the cognitive functions seemed to be normally distributed (Supplementary Figs. S1 and S2).

Another limitation was that the follow-up assessments were conducted when it was convenient for the patients, leading to considerable variability in the time interval between the discharge and follow-up assessments. Furthermore, a ceiling effect is possible in relation to participants achieving high neuropsychological assessment scores at admission, where the tests at discharge may not have been able to identify improvement.

Although our patients significantly improved their weight during partial weight restoration treatment and medical stabilization, they were still severely malnourished at discharge. Furthermore, the sample size should be considered when interpreting the non-significant association results from admission to discharge. While the intended sample size of 25 participants should be sufficient to identify plausible data patterns [42], it might not have sufficient power to detect an existing association at the given significance level. Furthermore, only 22 of the expected 25 participants completed the discharge assessment. High dropout is a well-known issue in research and interventions for patients with AN, also among patients with less severe AN than the patients in the current study [54]. However, the dropout rate in the current study was higher than the expected 20%, especially in relation to the follow-up assessment conducted after discharge. There were no baseline differences between patients who completed discharge and patients who dropped out before discharge.

The frequency of diagnosed comorbidity was relatively low in the current sample compared to several published studies of comorbidity in patients with AN of long duration. We excluded patients with comorbid schizophrenia or abuse but did not systematically screen for psychiatric comorbidity as this was not a study objective. This could explain the apparent low frequency of personality disorder, obsessive compulsive disorder, autism, and depression in our sample. Many of our patients had been severely malnourished for years, making a diagnosis of psychiatric comorbidity challenging. We recently confirmed that depressive symptoms decreased alongside reduced eating disorder psychopathology during treatment [55].

### Supplementary Information

Below is the link to the electronic supplementary material.

Supplementary file1 (DOCX 617 KB)

## Data Availability

The data that support the findings of this study is available in an anonymized version from the corresponding author upon reasonable request. The materials used are licensed (references in text).
