# Supplementary material for: Cognitive performance in hospitalized patients with severe or extreme anorexia nervosa
Source: Eat Weight Disord. 2023 Oct 21;28(1):86. doi: 10.1007/s40519-023-01585-w (PMC10590307; doi:10.1007/s40519-023-01585-w)
Supplement: Supplementary file 1 — Supplementary file1 (DOCX 617 KB) [file 40519_2023_1585_MOESM1_ESM.docx]

**Supplementary materials**

Supplementary Figure 1 illustrates the sample distribution of the WMS-III and the WAIS-IV index scores of the study sample compared to the normal distribution curves of the normative data.

Supplementary Figure 2 illustrates the sample distribution of the switching conditions of the Trail Making Test, Design Fluency Test, and the Verbal Fluency Test of the study sample compared to the normal distribution curves of the normative data.

Figure 1. Histograms of the WMS-III and the WAIS-IV indexes for the study sample compared to normal distribution curves of the normative data (grey= immediate indexes; purple=delayed indexes; rose=recognition delayed index; green=general memory index; light blue= working memory index; darker blue=processing speed index).

Figure 2. Histograms of the switching conditions on the Trail Making Test, the Design Fluency Test, and the Verbal Fluency Test for the study sample compared to normal distribution curves of the normative data.

**Supplementary Table 1. The relationship between proportional weight change and change in cognitive performance on D-KEFS tasks raw scores in patients with severe anorexia nervosa from admission (T_0_) to discharge (T_1_) and from discharge (T_1_) to follow-up (T_2_).**

| **Verbal Fluency Test raw scores** | | | | | | |
| --- | --- | --- | --- | --- | --- | --- |
|  | **Condition 1: Phonemic Fluency** | | **Condition 2: Semantic fluency** | | **Condition 3: Category switching, responses** | |
|  | **β (95% CI)** | ***p*** | **β (95% CI)** | ***p*** | **β (95% CI)** | ***p*** |
| **T_0_** ® **T_1_** | 0.325  (-0.265-0.915) | 0.263 | -0.142  (-0.660-0.376) | 0.572 | -0.055  (-0.346-0.235) | 0.694 |
| **T_1_**® **T_2_** | -0.051  (-0.342-0.239) | 0.707 | -0.122  (-0.346-0.102) | 0.259 | 0.015  (-0.105-0.135) | 0.792 |
|  | **Condition 3: Category switching, switching** | | **Repetition errors** | | **Category errors** | |
|  | **β (95% CI)** | ***p*** | **β (95% CI)** | ***p*** | **β (95% CI)** | ***p*** |
| **T_0_** ® **T_1_** | 0.022  (-0.299-0.344) | 0.886 | 0.057  (-0.085-0.199) | 0.410 | -0.070  (-0.154-0.013) | 0.095 |
| **T_1_**® **T_2_** | 0.001  (-0.114-0.117) | 0.980 | -0.019  (-0.072-0.033) | 0.438 | -0.013  (-0.052-0.026) | 0.479 |

| **Design Fluency Test raw scores** | | | | | | |
| --- | --- | --- | --- | --- | --- | --- |
|  | **Condition 1: Filled dots** | | **Condition 2: Empty dots** | | **Condition 3: Switching** | |
|  | **β (95% CI)** | ***p*** | **β (95% CI)** | ***p*** | **β (95% CI)** | ***p*** |
| **T_0_** ® **T_1_** | -0.044  (-0.210-0.122) | 0.584 | -0.085  (-0.353-0.182) | 0.513 | 0.071  ( -0.069-0.210) | 0.303 |
| **T_1_**® **T_2_** | -0.040  (-0.131-0.052) | 0.369 | 0.021  (-0.071-0.113) | 0.626 | 0.094  (0.016-0.172) | 0.021 |
|  | **Repetition errors** | | **Category errors** | |  |  |
|  | **β (95% CI)** | ***p*** | **β (95% CI)** | ***p*** |  |  |
| **T_0_** ® **T_1_** | -0.122  (-0.387-0.144) | 0.349 | -0.082  (-0.178-0.013) | 0.087 |  |  |
| **T_1_**® **T_2_** | -0.061  (-0.211-0.090) | 0.401 | -0.036  (-0.092-0.020) | 0.190 |  |  |

| **Trail Making Test raw scores** | | | | | | |
| --- | --- | --- | --- | --- | --- | --- |
|  | **Condition 1: Visual search** | | **Condition 2: Numbers** | | **Condition 3: Letters** | |
|  | **β (95% CI)** | ***p*** | **β (95% CI)** | ***p*** | **β (95% CI)** | ***p*** |
| **T_0_** ® **T_1_** | 0.102  (-0.237-0.440) | 0.537 | -0.125  (-0.544-0.295) | 0.541 | 0.115  (-0.397-0.626) | 0.644 |
| **T_1_**® **T_2_** | -0.071  (-0.230-0.086) | 0.345 | 0.000  (-0.259-0.261) | 0.998 | -0.211  (-0.442-0.021) | 0.072 |
|  | **Condition 4: Number-letter** | | **Condition 4: Errors** | | **Condition 5: Motor speed** | |
|  | **β (95% CI)** | ***p*** | **β (95% CI)** | ***p*** | **β (95% CI)** | ***p*** |
| **T_0_** ® **T_1_** | -0.032  (-1.831-1.766) | 0.970 | -0.005 (-0.082-0.073) | 0.903 | -0.070  (-0.405-0.266) | 0.669 |
| **T_1_**® **T_2_** | -0.361  (-1.111-0.388) | 0.317 | 0.006  (-0.022-0.034) | 0.645 | -0.065  (-0.273-0.143) | 0.509 |
| CI=Confidence interval  *Note.* Šidák corrected significance level: α=0.0015. | | | | | | |

**Supplementary Table 2. The relationship between proportional weight change and change in cognitive performance on d2-R raw scores in patients with severe anorexia nervosa from admission (T_0_) to discharge (T_1_) and from discharge (T_1_) to follow-up (T_2_).**

| **d2-R raw scores** | | | | | | |
| --- | --- | --- | --- | --- | --- | --- |
|  | **Processed targets** | | **Errors** | | **Accuracy (% errors)** | |
|  | **β (95% CI)** | ***p*** | **β (95% CI)** | ***p*** | **β (95% CI)** | ***p*** |
| **T_0_** ® **T_1_** | -0.342  (-3.665-2.981) | 0.832 | -0.091  (-1.001-0.819) | 0.836 | 0.021  (-0.148-0.191) | 0.794 |
| **T_1_**® **T_2_** | -0.159  (-1.512-1.195) | 0.804 | 0.116  (-.216-0.447) | 0.464 | 0.021  (-0.044-0.086) | 0.495 |
|  | **Corrected total score** | | **Concentration performance** | |  |  |
|  | **β (95% CI)** | ***p*** | **β (95% CI)** | ***p*** |  |  |
| **T_0_** ® **T_1_** | -0.222  (-2.963-2.520) | 0.867 | -0.164  (-1.379-0.050) | 0.780 |  |  |
| **T_1_**® **T_2_** | -0.020  (-1.300-1.260) | 0.974 | -0.143  (-0.858-0.571) | 0.672 |  |  |
| CI=Confidence interval  *Note.* Šidák corrected significance level: α=0.0051. | | | | |  |  |

**Supplementary Table 3. The relationship between proportional weight change and change in cognitive performance on WMS-III and WAIS-IV indexes in patients with severe anorexia nervosa from admission (T_0_) to discharge (T_1_) and from discharge (T_1_) to follow-up (T_2_).**

| **WMS-III indexes** | | | | | | | | | **WAIS-IV index** | |
| --- | --- | --- | --- | --- | --- | --- | --- | --- | --- | --- |
|  | **Auditory**  **immediate index** | | **Visual**  **immediate index** | | **Immediate**  **memory index** | | **Auditory delayed index** | | **Processing speed index** | |
|  | **β (95% CI)** | ***p*** | **β (95% CI)** | ***p*** | **β (95% CI)** | ***p*** | **β (95% CI)** | ***p*** | **β (95% CI)** | ***p*** |
| **T_0_** ® **T_1_** | -0.113  (-0.758-0.532) | 0.718 | 0.258  (-0.683-1.199) | 0.573 | 0 .073  (-0.795-0.941) | 0.862 | 0.010  (-0.697-0.718) | 0.976 | -0.080  (-0.814-0.653) | 0.821 |
| **T_1_** ® **T_2_** | -0.046  (-0.396-0.303) | 0.779 | -0.311  (-0.580-(-0.042)) | 0.027 | -0.249  (-0.543-0.045) | 0.091 | -0.009  (-0.411-0.393) | 0.962 | 0.009  (-0.274-0.291) | 0.949 |
|  | **Visual delayed index** | | **Auditory Recognition Delayed Index** | | **General memory index** | | **Working memory index** | |  | |
|  | **β (95% CI)** | ***p*** | **β (95% CI)** | ***p*** | **β (95% CI)** | ***p*** | **β (95% CI)** | ***p*** |  | |
| **T_0_** ® **T_1_** | -0.092  (-1.113-0.930) | 0.853 | -0.461  (-1.545-0.623) | 0.384 | -0.159  (-0.933-0.615) | 0.672 | 0.476  (-0.140-1.092) | 0.123 |  | |
| **T_1_** ® **T_2_** | 0.083  (-0.354- | 0.689 | -0.201  (-0.702-0.299) | 0.400 | 0.028  (-0.369-0.427) | 0.879 | 0.088  (-0.175-0.351) | 0.481 |  | |
| CI=Confidence interval  *Note.* Šidák corrected significance level: α=0.0028 | | | | | | | | | | |
